# Evaluation of the short‐term echocardiographic effects of two loop diuretics, furosemide and torsemide, in a group of dogs

**DOI:** 10.1002/vms3.1129

**Published:** 2023-05-30

**Authors:** Farzaneh Hosseini, Zeinab Mahmoudi Filabadi, Peter B Hill, Morteza Hosseininejad

**Affiliations:** ^1^ Faculty of Veterinary Medicine, Department of Clinical Sciences Shahrekord University Shahrekord Iran; ^2^ School of Veterinary and Animal Sciences The University of Adelaide Adelaide Australia

**Keywords:** blood pressure, canine, diuretics, echocardiography, frusemide, torsemide

## Abstract

**Background:**

Two loop diuretics, torsemide and frusemide, can affect the urinary system and consequently the cardiordiovascular haemodynamics in different ways.

**Objectives:**

This study compared a number of echocardiographic parameters and systemic arterial blood pressure (ABP) changes following administration of furosemide or torsemide.

**Methods:**

Five shelter dogs underwent transthoracic two‐dimensional M‐mode echocardiography to obtain the following measurements: left ventricular internal dimension at end‐systole (LVIDs), left ventricular internal dimension at end‐diastole (LVIDd), fractional shortening (FS), heart rate (HR) and the distance between the mitral valve socket and the ventricle wall (septal to E Point, SEP). Arterial blood pressure was measured using the oscillometric method. Measurements recorded before treatment (baseline data) were compared to those after the dogs received furosemide (5 mg/kg) or torsemide (0.5 mg/kg).

**Results:**

Torsemide significantly reduced blood pressure 1 h after administration, but this was not seen with furosemide. Fractional shortening, LVIDd and SEP decreased following both treatments, but there were no significant differences between the treatment groups. Torsemide increased heart rate above that seen in the furosemide groups.

**Conclusions:**

The results of this study indicate that 1 h after administration, torsemide increases heart rate and decreases blood pressure when compared to furosemide, but both drugs have similar effects on measured cardiovascular indices.

## Graphical abstract

Cardiovascular effects of torsemide and frusemide were compared in dogs. Torsemide was more potent in decreasing blood pressure. Cardiovascular indices were the same for these two drugs.

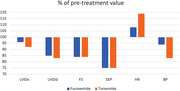

## 1 INTRODUCTION

Diuretics are among the most commonly used drugs for medical management of congestive heart failure (CHF) (Atkins et al., 2009). Three types of diuretics are commonly used in veterinary medicine: thiazides, loop diuretics and potassium‐sparing diuretics. The most important effect of these drugs is to induce water loss via the kidneys. This reduces blood volume, which can affect cardiovascular function and haemodynamics, such as a decrease in diastolic chamber size, an increase in myocardial wall thickness, reduced cardiac output and tissue perfusion (Campbell & Kittleson, 2007; Di Segni et al., 1997; Prisant et al., 1994).

Loop diuretics are the most potent and most commonly used diuretics in human and veterinary medicine. For many years, furosemide has been the most frequently used loop diuretic (Atkins et al., 2009; Bikdeli et al., 2013) but more recently torsemide has been introduced as a more attractive candidate for CHF treatment. Recent studies indicated that the quality of life and survival increased in humans receiving torsemide instead of furosemide (Cosín & Díez, 2002; Mentz et al., 2016). Torsemide is more potent than furosemide and leads to greater diuresis and natriuresis, while possibly less kaliuresis (Ghys et al., 1985; Sogame et al., 1996). In dogs, a furosemide dose between 1 and 5 mg/kg has been recommended for the treatment of acute and chronic heart failure (Ramsey, 2017). In comparison, torsemide was shown to be 10 times more potent than furosemide when their diuretic effects were compared (Uechi et al., 2003). In another study, a similar diuresis was obtained when torsemide was administered to dogs at 1/20th the dosage of furosemide over a 10‐day period, but the degree of activation of the renin‐angiotensin aldosterone system (RAAS) did not differ significantly between the two (Potter et al., 2019).

The greater diuretic effect of torsemide is due to a long duration of action, when compared to the rapid and transient induction of urine production caused by furosemide (Hori et al., 2007). For these reasons, torsemide is recommended for the treatment of CHF in humans (Vargo et al., 1995).

Despite various studies performed on the haemodynamic impacts of these two drugs in dogs (Chetboul et al., 2017; Hori et al., 2007), to our knowledge, the comparative effects of torsemide and furosemide on the canine cardiovascular system have not been previously evaluated. We hypothesised that different effects of torsemide and furosemide on the urinary system could affect the cardiovascular system as well and, consequently, echocardiographic parameters . Accordingly, in this study, the effects of furosemide and torsemide on the cardiovascular system, blood pressure and echocardiographic parameters were compared.

## 2 MATERIALS AND METHODS

Five clinically healthy, crossbreed, 1‐ to 2‐year‐old dogs were used in this study. These dogs were housed in a shelter before being entered into the study. The research was conducted in the small animal clinic of Shahrekord University where the dogs were housed in separate cages under the same environmental and nutritional conditions for 2 weeks before the study. They were dewormed using praziquantel (a single dose of 5 mg/kg) and mebendazole (20 mg/kg for 5 days) 14 days before starting the trial. Each dog was also vaccinated against rabies.

Echocardiography was performed on the five dogs before receiving any treatment to establish baseline values and then 1 h after receiving a single dose of furosemide at a dose of 5 mg/kg. Echocardiography was repeated 1 week later, 1 h after the dogs were given a single dose of torsemide at a dose of 0.5 mg/kg. During this week of wash out period, dogs did not receive any medications.

To perform echocardiography, the right side of the chest was completely shaved between the 3rd to 6th intercostal spaces. Initially, by using the two‐dimensional B‐mode method, echocardiographic images were obtained from the right approach in the longitudinal axis. After identifying and evaluating the cardiac structures, transverse images were obtained by 90° transducer rotation, and the following measurements were obtained using M‐mode: left ventricular internal dimension at end‐systole (LVIDs), the left ventricular internal dimension at end‐diastole (LVIDd), fractional shortening (FS), heart rate (HR), and septal to E point (SEP). To measure LVIDs, the distance between the two peaks was measured in the M‐mode images, and the distance between the two near‐dips was measured to obtain the LVIDd. FS was calculated as ([LVIDd−LVIDs]/LVIDd) × 100 (Visser et al., 2019). Each measurement was performed 5–6 times and averaged.

Systolic blood pressure and heart rate were measured before and approximately 1 h after administration of each drug using the oscillometric method (Biosys Guardian BPM 700, Korea) and a cuff size that was approximately 40% the circumference of the cuff site (Brown et al., 2007).

Collected data were categorised into three data groups: baseline, frusemide and torsemide. The baseline data group was the mean ± SD of each measured parameter before giving either of the two medications. Baseline data (before treatment) were compared to posttreatment data using one‐way analysis of variance (one‐way ANOVA) and differences were considered significant when *p* was less than 0.05. SPSS‐24 software was used for data analysis.

## 3 RESULTS

The baseline and posttreatment cardiac and echocardiographic data are shown in Tables 1 and 2. Treatment with furosemide resulted in a significant decrease in fractional shortening (*p* = 0.047), SEP (*p* = 0.007) and LVIDd (*p* = 0.009). No significant changes were seen in HR, BP or LVIDs.

**TABLE 1:** echocardiographic indices following prescription of furosemide or torsemide in dogs.

Groups	LVIDs	LVIDd	HR	FS	SEP
	(mean ± SD)	(mean ± SD)	(mean ± SD)	(mean ± SD)	(mean ± SD)
Baseline data (F)	2.50 ± 0.1 ^*^	4.11 ± 0.12 ^†^	89 ± 3 ^¥^	37.49 ± 3 ^¶^	0.71 ± 0.03 ^#^
Furosemide	2.4 ± 0.06 ^*^	3.52 ± 0.07 ^††^	95.7 ± 2.58 ^¥^	31.43 ± 0.96 ^¶¶^	0.53 ± 0.02 ^##^
Baseline data (T)	2.54±0.1 ^*^	4.11±0.12 ^†^	87±3 ^¥^	37.4±3 ^¶^	0.71 ± 0.03 ^#^
Torsemide	2.32 ± 0.03 ^*^	3.41 ± 0.06 ^††^	105.6 ± 2.02 ^¥¥^	31.15 ± 0.86 ^¶¶^	0.53 ± 0.02 ^##^

Baseline data were measured for frusemide (F) and torsemide (T). Different asterisks indicate significant differences between data related to measured indices.

One specific symbol is allocated to each parameter (^*^ for LVIDs, ^†^ for LVIDd, ^¥^ for HR, ^¶^ for FS, ^¶¶^ for SEP). All the baseline data have only one single symbol (^*^). When the baseline data is not significantly different from Frusemide (or Torsemide) the latter data will also gain a single symbol (^*^) but if they are significantly different from the baseline data, they will gain 2 symbols (^**^).

**TABLE 2:** Effects of furosemide or torsemide on systolic blood pressure (mean ± SD) before and 1 h after administration of the drug.

Groups	Before administration (baseline data)	1 h after administration
Furosemide	123.2 ± 7.32^*^	116. 2 ± 4.63 ^†^
Torsemide	122. 2 ± 5.22^*^	102.2 ± 4.75 ^††^

Different asterisks indicate significant differences between data related to measured indices.

^*,†^ means no significant difference between them.

^††^ means that these two data are different significantly.

Treatment with torsemide also resulted in a significant decrease in fractional shortening (*p* = 0.031), SEP (*p* = 0.005) and LVIDd (*p* = 0.001), but there was also a significant increase in HR (*p* = 0.016) and a significant decrease in BP (*p* = 0.022).

When the posttreatment effects of furosemide and torsemide were directly compared, there was no significant difference between the FS (*p* = 0.84), SEP (*p* = 0.84), LVIDd (*p* = 0.28) or LVIDs (*p* = 0.45), but the HR was significantly higher following torsemide treatment (*p* = 0.007) and the BP was significantly lower.

**FIGURE 1:**
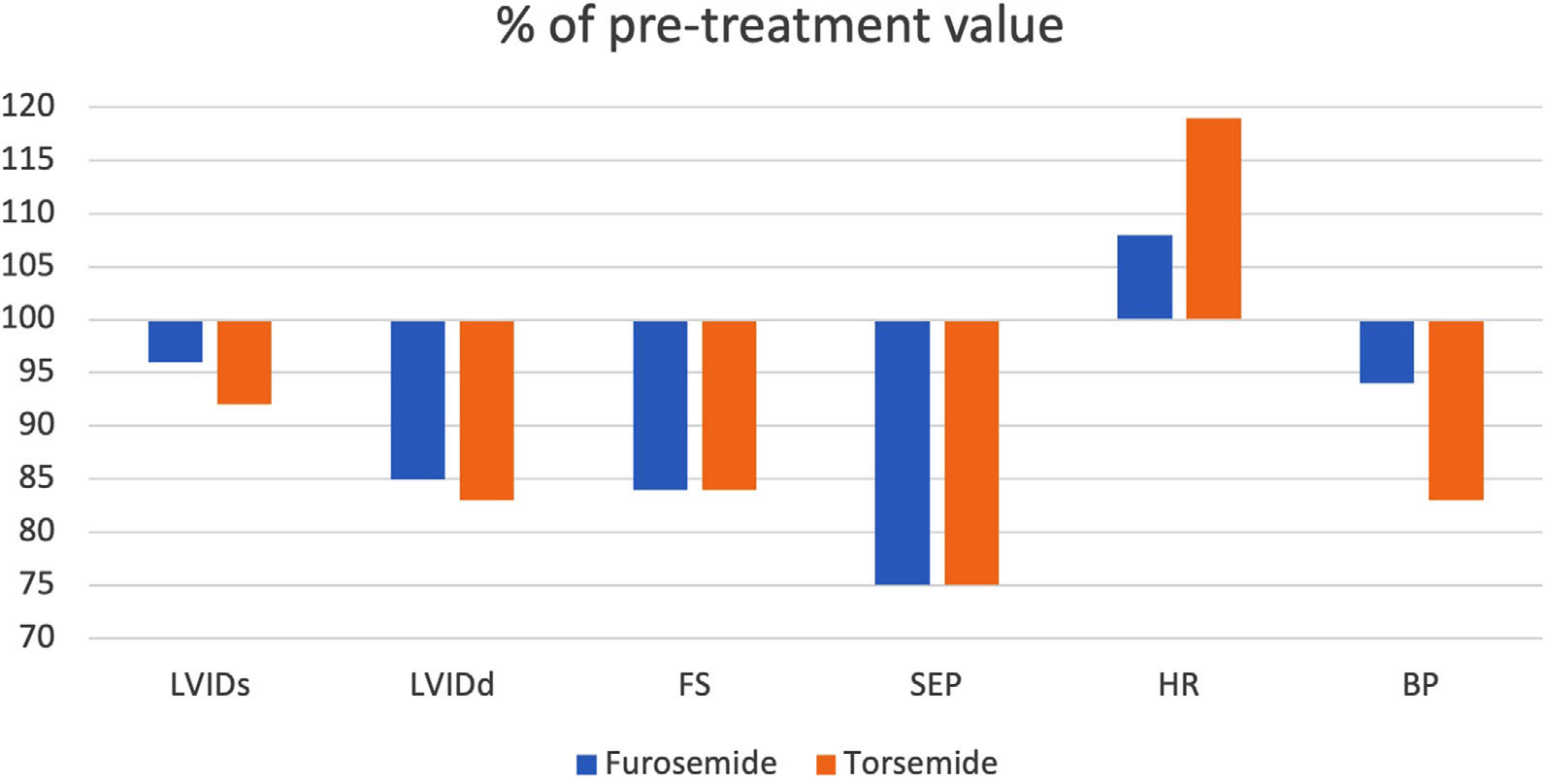
Percentage of measured factors to their relevant pretreatment values.

The percentages of the echocardiographic and cardiologic changes compared to pretreatment parameters are depicted in Figure 1.

## 4 DISCUSSION

Torsemide, a newer loop diuretic, is commonly included as a treatment for human CHF together with other medications of different classes (Müller et al., 2003). Based on the ACVIM consensus guidelines for the diagnosis and treatment of myxomatous mitral valve disease in dogs, torsemide is recommended as a substitute for furosemide for chronic (home‐based) treatment at approximately 5%–10% of the furosemide dosage q24h (Hori et al., 2007; Keene et al., 2019).

One of the important steps in the treatment of cardiovascular disease is to control systemic blood pressure, because sustained hypertension causes injury to tissues (Acierno et al., 2018). Blood pressure can be measured using different methods, with direct BP measurement providing the most accurate data. However, this is rarely practical and oscillometric BP measurement is more representative of what is used in clinical practice. Accordingly, in this study we used oscillometric BP measurement (Stepien et al., 2003). A number of different medications are available to treat hypertension in dogs including angiotensin converting enzyme inhibitors, angiotensin receptor blockers, calcium channel blockers, alpha‐1 blockers, direct vasodilators, aldosterone antagonists, beta blockers and diuretics (thiazide and loop diuretics) (Keene et al., 2019). Although diuretics are not a first‐line treatment for decreasing blood pressure in dogs, an antihypertensive effect alongside a diuretic effect could be valuable in cases of congestive heart failure. In our study, torsemide had a greater effect on blood pressure than furosemide following short‐term treatment, suggesting it could be beneficial in cases of canine congestive heart failure accompanied by hypertension. However, this study was performed on a limited number of healthy dogs, and a more comprehensive clinical trial would be required to verify this benefit in clinically unwell cases.

Administration of both furosemide and torsemide led to a reduction in fractional shortening. Fractional shortening represents the systolic performance of the heart. One of the physiological mechanisms related to FS is that if the left ventricle is not completely filled at the end of diastole, the FS will reduce. Torsemide significantly increased the heart rate compared to furosemide and nontreated dogs. Following an increase in heart rate, there will not be enough time for the heart to refill, and the left ventricular end‐diastolic volume (LVEDV) decreases and generally leads to a reduction in the FS. However, if this moderate increase in the heart rate can actually affect FS directly, needs to be investigated in a bigger study group. In terms of the echocardiographic parameters, decreased LVIDd is followed by diminished diastolic end points due to decreased blood volume and reduced cardiac preload. It is indicated that fractional shortening is influenced by many parameters including preload, afterload and contractility, all of which may act independently or in combination to affect it (Gugjoo et al., 2014). According to the results, there are no differences between the measured LVIDs, LVIDd and SEP parameters following using either of these two diuretics and they are expected to have the same echocardiographic characteristics.

## 5 CONCLUSION

The results of this study indicate that the use of torsemide leads to a greater reduction in blood pressure than furosemide, while both drugs have similar impacts on the other measured cardiovascular parameters. In this study, only a single dose of each diuretic in a few dogs was used and a long‐term study using different dosages of these diuretics in a larger group is required to perform a more comprehensive comparison between the effects of torsemide and frusemide. Moreover, absorption of frusemide and torsemide may be affected by poor gastrointestinal perfusion due to heart failure and findings of this study cannot be applied to clinical patients.

## AUTHOR CONTRIBUTIONS

Farzaneh Hosseini: investigation; supervision; writing – review & editing. Morteza Hosseininejad: funding acquisition; investigation; methodology; project administration; supervision; writing – original draft; writing – review & editing.

## CONFLICT OF INTEREST STATEMENT

The authors declare no conflicts of interest.

### ETHICS STATEMENT

Because the medications used in this study revealed to be safe for the dogs in previous studies, and based on the *guide for the care and use of laboratory animals* (Chetboul et al., 2017; Council, 1996), animal ethics could be approved by the research committee of Shahrekord University (Approval No. 332178‐26/2/96).

### PEER REVIEW

The peer review history for this article is available at https://publons.com/publon/10.1002/vms3.1129

## Data Availability

Research data are available.
